# Trapped in a Glass Bell Jar: Neural Correlates of Depersonalization and Derealization in Subjects at Clinical High-Risk of Psychosis and Depersonalization–Derealization Disorder

**DOI:** 10.3389/fpsyt.2020.535652

**Published:** 2020-09-11

**Authors:** Jessica R. Büetiger, Daniela Hubl, Stephan Kupferschmid, Frauke Schultze-Lutter, Benno G. Schimmelmann, Andrea Federspiel, Martinus Hauf, Sebastian Walther, Michael Kaess, Chantal Michel, Jochen Kindler

**Affiliations:** ^1^ University Hospital of Child and Adolescent Psychiatry and Psychotherapy, University of Bern, Bern, Switzerland; ^2^ University Hospital of Psychiatry and Psychotherapy, University of Bern, Bern, Switzerland; ^3^ Integrated Psychiatric Services of Winterthur and Zurich Unterland (ipw), Winterthur , Switzerland; ^4^ Department of Psychiatry and Psychotherapy, Medical Faculty, Heinrich-Heine-University, Düsseldorf, Germany; ^5^ University Hospital of Child and Adolescent Psychiatry, University Hospital Hamburg-Eppendorf, Hamburg, Germany; ^6^ Support Center for Advanced Neuroimaging (SCAN), Institute for Diagnostic and Interventional Neuroradiology, University of Bern, Bern, Switzerland; ^7^ Section for Translational Psychobiology in Child and Adolescent Psychiatry, Department of Child and Adolescent Psychiatry, Center for Psychosocial Medicine, University of Heidelberg, Heidelberg, Germany

**Keywords:** clinical high risk for psychosis, depersonalization, derealization, arterial spin labeling, magnetic resonance imaging, orbitofrontal cortex, caudate nucleus

## Abstract

**Background:**

Depersonalization (DP) and derealization (DR) are symptoms of a disruption of perceptual integration leading to an altered quality of subjective experiences such as feelings of unreality and detachment from the self (DP) or the surroundings (DR). Both DP and DR often occur in concert with other symptoms, for example in subjects at clinical high-risk (CHR) for psychosis, but also appear isolated in the form of DP/DR disorder. Despite evidence that DP/DR causes immense distress, little is known about their neurobiological underpinnings. Therefore, we investigated the neural correlates of DP/DR using pseudo-continuous arterial spin labeling MRI.

**Methods:**

We evaluated the frequency of DP/DR symptoms in a clinical sample (N = 217) of help-seeking individuals from the Early Detection and Intervention Centre for Mental Crisis (CHR, n = 97; clinical controls (CC), n = 91; and first-episode psychosis (FEP), n = 29). Further, in a subsample of those CHR subjects who underwent MRI, we investigated the resting-state regional cerebral blood flow (rCBF). Here, individuals with (n = 21) and without (n = 23) DP/DR were contrasted. Finally, rCBF was measured in a small independent second sample of patients with DP/DR disorder (n = 6) and healthy controls (HC, n = 6).

**Results:**

In the complete clinical sample, significantly higher frequency of DP/DR was found in CHR compared to CC (50.5 *vs.* 16.5%; χ^2^_(2)_ = 24.218, p ≤ 0.001, Cramer’s V = 0.359) as well as in FEP compared to CC (37.9 *vs.* 16.5%; χ^2^_(2)_ = 5.960, *p* = 0.015, Cramer’s V = 0.223). In MRI, significantly lower rCBF was detected in the left orbitofrontal cortex in CHR with *vs.* without DP/DR (x/y/z = −16/42/−22, p < 0.05, FWE corrected). In patients with DP/DR disorder, significantly higher rCBF was detected in the left caudate nucleus (x/y/z = −18/−32/18, p < 0.05) compared to HC.

**Conclusions:**

This study shows that DP/DR symptoms are frequently found in CHR subjects. Investigating two separate DP/DR populations with an identical neuroimaging technique, our study also indicates that there may be divergent pathophysiological mechanisms—decreased neuronal activity in the orbitofrontal cortex, but increased activity within the caudate nucleus—leading to a final common pathway with similar psychopathological symptoms. This suggests that both top-down (orbitofrontal cortex) and bottom-up (caudate nucleus) mechanisms could contribute to the emergence of DP/DR.

## Introduction

Altered subjective experiences such as feelings of unreality and detachment from the self or the surroundings are defined as depersonalization (DP) and derealization (DR). Individuals may feel detached from the whole self or from aspects of the self, including feelings, thoughts, body parts or sensations, and from individuals, objects, or all surroundings, often described as being in a fog, dream or bubble, being numb, or as if they are under a glass bell (1–4). DP/DR is a ‘physiological’ perceptual reaction and psychological phenomenon, especially occurring when stressed, but also when traumatized, very tired, anxious, or intoxicated, however, with sustained insight into the subjective nature of the symptoms. In most cases, these DP/DR experiences are transient, but in some cases, DP/DR may take a chronic course, persisting for days, weeks, or months, with episodic or permanent symptoms. Individuals with DP/DR frequently worry about their mental state and are frightened of becoming crazy or losing their mind (1, 5). DP/DR disorder is characterized by a persistent or recurrent experience of unreality and detachment from oneself or the surrounding, while reality testing remains intact. It is a primary mental health disorder and occurs in the absence or only secondary development of other mental disorders. Symptoms result in significant distress or impairment in functioning (5, 6).

Common age of onset is adolescence, with earlier onset associated with higher severity and poorer prognosis (3, 7). In a systematic review, a prevalence rate of 1.2–2.4% was found for clinically significant DP/DR symptoms in the community and 30–82% in clinical samples (1). DP/DR disorder is often seen in clinical conditions as comorbidity, especially in psychoses (1, 8, 9), depression and anxiety disorders (7), and also after cannabis abuse (10).

Depending on the setting, a review reported DP/DR symptom rates from 7% in outpatients to 36% in inpatients with manifest psychosis (1). Patients with manifest psychosis had higher DP/DR scores according to the ‘Cambridge Depersonalization Scale’ (CDS) (11) compared to first-degree relatives and healthy controls (12). Furthermore, patients with manifest psychosis were assessed for DP with the ‘Bonn Scale for the Assessment of Basic Symptoms’ (13) and could be differentiated from those without DP with the paradigm of Basic Symptoms (14). Basic symptoms are subtle, subclinical, self-experienced disturbances in drive, stress tolerance, affect, thinking, speech, perception and motor action. They are experienced with full insight into their abnormal nature. Basic symptoms can be present before, during, and after psychotic episodes (15). Additionally, DP/DR was found to be more frequent, had a longer duration and was stronger in the early, compared to the chronic stages, of psychosis (8). Furthermore, DP/DR symptoms were also reported to occur in ultra-high risk (UHR) subjects for psychosis (16, 17).

The majority of first-episode psychotic disorders are preceded by a prodromal phase in which a multitude of CHR symptoms (including DP/DR), other mental health problems, and psychosocial deficits occur, and during which help may be sought (18–20). This phase offers an excellent starting point for an indicated prevention that aims at reducing CHR symptoms and, thereby, preventing transition to frank psychosis (19). Currently, two major sets of CHR criteria are used to detect a putatively psychosis-prodromal phase: (i) symptomatic ultra-high risk (UHR) criteria, *i.e.*, attenuated (APS) or brief intermittent psychotic symptoms (BIPS); and (ii) basic symptom criteria, *i.e.*, Cognitive Disturbances (COGDIS) and Cognitive-Perceptive Basic Symptoms (COPER) (18, 20).

As DP/DR experiences can occur on a continuum from transient symptoms to chronic ones (1), the question arises whether the symptoms are caused by similar or distinct pathophysiological mechanisms in different clinical groups/diagnoses. Previous studies suggest that different systems such as the fronto-limbic and the temporo-parietal network are responsible for different DP/DR symptoms (21). The theory of the fronto-limbic system proposes that the frontal cortex activity is increased while the limbic system is inhibited (*e.g.* amygdala) causing a reduction of emotional responses (*e.g.* numbing, perceptual detachment) (22), whereas the temporo-parietal system could lead to the emergence of feelings of disembodiment and lack of agency seen in DP/DR patients (21). Different studies confirmed the involvement of both systems with DP/DR across diverse examination methods such as whole brain magnetic resonance imaging (MRI), fractional anisotropy, and positron emission tomography (23–26).

Further, symptom improvement of DP/DR showed altered insula, visual cortex, and cerebellum activation (27). Treatment of DP/DR with repetitive transcranial magnetic stimulation in patients with DP/DR disorder showed that inhibition applied to the ventrolateral prefrontal cortex and temporo-parietal junction leads to symptom reductions in DP/DR, indicating that both systems have associations with DP/DR (28, 29). Besides the fronto-limbic and the temporo-parietal system, the striatum was also linked to DP/DR (30). These findings, together with the findings of decreased gray matter in the right caudate, right thalamus, and right cuneus as well as of gray matter increases in the left dorsomedial prefrontal cortex and right somatosensory region (23), and alterations in white matter in the left caudate nucleus, the right amygdala and brainstem (24) point towards dysfunctions in different systems.

Taken together, DP/DR symptoms might be linked to brain regions involving the frontal-limbic and temporo-parietal network as well as the striatum (2, 4, 21, 30, 31). For a better understanding of the neuronal mechanisms underlying DP/DR, we used resting state cerebral blood flow (rCBF), a proxy for localized neuronal activity that can be measured with arterial spin labeling (ASL)-MRI (32–34). ASL-MRI measures perfusion using magnetically labeled arterial blood as a tracer. Thereby, ASL-MRI provides a quantifiable measure of regional cerebral blood flow (in ml/min/100 g brain tissue) reflecting the level of glucose metabolism which is associated with neuronal activity of the respective cerebral area (34, 35). We have previously used ASL to successfully capture specific psychopathological symptoms (36–40).

Our aim was to evaluate the frequency of DP/DR symptoms in CHR subjects in a first step and—in a second step—to investigate the neuronal correlates of DP/DR symptoms using the same neuroimaging method in two different clinical samples. First, we assessed the frequency of DP/DR in help-seeking subjects at an early detection service. Second, we assessed rCBF with ASL-MRI in CHR subjects with and without DP/DR symptoms (sample 1). Third, we compared rCBF of a small sample fulfilling criteria for DP/DR disorder with healthy controls (sample 2). We expected to find a high frequency of DP/DR symptoms in CHR and, associated with DP/DR, rCBF alterations in frontal, temporal, or striatal areas in both samples.

## Materials and Methods

### Sample and Assessments

Two independent samples were assessed for this study. The first sample (sample 1) was recruited at the Bern Early Recognition and Intervention Center for Mental Crisis (FETZ Bern; www.upd.ch/de/angebot/erwachsenenpsychiatrie/ambulant-fetz.php) between November 2009 and June 2018. Individuals with various psychiatric symptoms were admitted to the FETZ Bern by physicians, psychosocial institutions, or of their own initiative, whenever there was clinical suspicion for a developing psychotic disorder. The second sample (sample 2) was recruited at the University Hospital of Psychiatry and Psychotherapy in Bern between July 2011 and January 2013 and consisted of patients with DP/DR disorder, exclusively, as well as of healthy controls.

All procedures contributing to this work comply with the ethical standards of the relevant national and institutional committees on human experimentation and with the Helsinki Declaration of 1975, as revised in 2013. The human research ethics committee of the Canton Bern approved the study (ID PB_2016-01991, KEK-095/10). All participants gave informed consent, and in minors, parental informed consent was provided.

### Psychopathology Assessment of Sample 1

Data from 245 subjects who were examined in the FETZ Bern entered the analyses. The FETZ Bern is the only early detection and intervention center for psychosis in the Canton of Bern, Switzerland, with a catchment area of approximately 1.5 million inhabitants, screening ~80 patients/year (aged 8–40 years) according to the European Psychiatric Association (EPA) guidelines (19, 20). The basic assessment includes a psychopathological evaluation, a cognitive test battery, MRI, and blood screening. Individuals were diagnosed as clinical controls (CC), first episode psychosis (FEP), or CHR (41). CHR and related symptoms were assessed by trained psychologists using semi-structured interviews including the ‘Schizophrenia Proneness Instruments Child & Youth and Adult’ (SPI-CY and SPI-A) to evaluate basic symptoms (42, 43), the ‘Structured Interview for Prodromal Syndromes’ (SIPS) (44), and the rating from the Comprehensive Assessment of At-Risk Mental States (CAARMS earlier version than 2006) (45) to evaluate UHR and related symptoms.

The SPI-A and later the SPI-CY were developed based on the BSABS. They assess the same concepts and are semi-structured interviews. However, the SPI-CY/SPI-A assesses the symptoms on a quantitative 7-point rating scale, as opposed for the BSABS which rates the symptoms qualitatively for their presence or absence only (13, 46). For more information about the various assessments, we refer to the EPA guidelines (20).

DP/DR symptoms can be assessed either with the SIPS and/or the SPI-CY/SPI-A. The SIPS rates DP/DR items (P1 and N4) in a lifetime; however, we focused on symptoms measured *via* SPIA/SPICY as we aimed to capture present symptoms (symptoms that were present within the last three months). Therefore, DP/DR was assessed within the last three months using operationalized items from the SPI-CY/SPI-A. Derealization (SPI-CY: item B7; SPI-A: item O8) was assessed from age 13 onwards (requires self-reflection and higher metacognitive processes) and is defined as a change in how the person relates emotionally to the world, *i.e.* by an ‘as if’-feeling that the world is not real or of oneself being estranged from it while knowing at the same time that it is real, and they are a part of it (see **Supplementary Text S1** for a detailed description). Depersonalization is rated in the SPI-CY from age 13 onwards with the items somatopsychic depersonalization (B8.2), *i.e.*, feeling estranged from one’s own body and autopsychic depersonalization (C6), *i.e.*, feeling estranged from one’s own actions, feelings or emotions, in any case, while being fully aware that it is them. In the SPI-A, only somatopsychic depersonalization (F6) can be assessed (see **Supplementary Text S1**).

The SPI-CY/SPI-A ranks items on a severity scale according to the maximum frequency of their occurrence within the past three months ranging from ‘0’ (absent = symptom has not occurred in the past 3 months) to ‘6’ (extreme = symptom has occurred daily over sometime within the past 3 months). Symptoms may also be rated as ‘7’ (symptom has always been present in same severity; trait), ‘8’ (symptom is definitively present, but its frequency of occurrence is unknown), and ‘9’ (the presence of the symptom can neither be unambiguously ruled in nor out). To dichotomize subjects into either having DP/DR symptoms or not, subjects scoring in any of the items from 1 to 6 or 8 were rated as having DP/DR, while subjects with no symptoms (0), unclear symptoms (9), or symptoms as traits (7) were considered to have no DP/DR.

Due to five abortions of the clinical assessments, 13 persons being younger than 13 years and 10 missing values for DP/DR due to incomplete interviews, complete behavioral data was accessible from 217 subjects. The CC did not fulfill any CHR criterion nor did they have a history of past or present psychosis, but they were help-seeking individuals fulfilling other psychiatric diagnoses (see **Table 1**). FEP fulfilled a past or present psychosis and CHR subjects did not fulfill a past or present psychosis but the CHR criterion.

**Table 1:** Sociodemographic and clinical characteristics of the complete sample 1 (CHR, FEP, CC).

		Total		CHR		FEP		CC		Statistical values^a^
		N = 217		n = 97		n = 29		n = 91		
Age in years										
	mean ± SD	19.2 ± 4.6		18.8 ± 3.9		20.5 ± 6.4		19.2 ± 4.7		H=0.758, *p*=0.684, df=2, ϵ^2 =^ 0.004
	Median	17.8		17.6		18.5		17.8		
	Range	13–40		13–35		13-40		13-37		
SOFAS score										
	mean ± SD	61.0 ± 12.0		60.1 ± 11.0		55.1 ± 11.3		63.7 ± 12.7		H=10.841, *p*=**0.004**, df=2, ϵ^2 =^ 0.050^b^
	Median	61.0		61.0		55.0		65.0		
	Range	32–89		35–83		35-75		32-89		
Gender, male (n, %)		126	58.1	52	53.6	16	55.2	58	63.7	χ^2^_(2)_=2.093, *p*=0.351, V=0.098
Current partnership, yes (n, %)		48	22.1	17	17.5	9	31.0	22	24.2	χ^2^_(2)_=3.384, *p*=0.184, V=0.131
Nationality, Swiss (n, %)		189	87.1	86	88.7	23	79.3	80	87.9	χ^2^_(2)_=0.099, *p*=1.000, V=0.011
Highest education (n, %)										χ^2^_(4)_=3.399, *p*=0.463, V=0.149
	ISCED 1 (6 school years)	5	2.3	1	1.0	0	0.0	4	4.4	
	ISCED 2 (9–10 school years)	138	63.6	62	63.9	22	75.9	54	59.3	
	ISCED 3 (12–13 school years)	58	26.7	28	28.9	6	20.7	24	26.4	
Currently employed or in training/school (n, %)^c^		185	85.3	84	86.6	22	75.9	79	86.8	χ^2^_(2)_=0.301, *p*=0.920, V=0.026
Current alcohol misuse, present (n, %)		10	4.6	5	5.2	1	3.4	4	4.4	χ^2^_(2)_=0.197, *p*=1.000, V=0.012
Current drug misuse, present (n, %)		16	7.4	10	10.3	0	0.0	6	6.6	χ^2^_(2)_= 2.385, *p*=0.288, V=0.121
Any current ICD-10 diagnosis (n, %)										
	Any affective disorder (F30–F39)	74	34.1	44	45.4	8	27.6	22	24.2	χ^2^_(2)_=9.207, *p*=**0.010**, V=0.220
	Any anxiety disorder (F40–F41)	37	17.1	25	25.8	3	10.3	9	9.9	χ^2^_(2)_ =7.449, *p*=**0.021**, V=0.198
	Any eating disorder (F50)	3	1.4	2	2.1	0	0.0	1	1.1	χ^2^_(2)_=0.536, *p*=1.000, V=0.056
	OCD (F42)	12	5.5	5	5.2	1	3.4	6	6.6	χ^2^_(2)_ =0.373, *p*=0.908, V=0.042
	PTBSD (F43.1)	1	0.5	1	1.0	0	0.0	0	0.0	χ^2^_(2)_=1.669, *p*=1.000, V=0.075
	DP/DR disorder (F48.1)	18	8.3	12	12.4	0	0.0	6	6.6	χ^2^_(2)_=4.803, *p*=0.080, V=0.153
DP/DR symptoms, present (n, %)		75	34.6	49*	50.5	11	37.9	15*	16.5	χ^2^_(2)_=24.212, *p ≤* **0.001**, V=0.334
DP symptom, present (n, %)		29	13.4	18	21.2	5	19.2	6	6.8	χ^2^_(2)_=8.136, *p*=**0.016**, V=0.196
DR symptoms, present (n, %)		64	29.5	43*	44.3	10	34.5	11*	12.4	χ^2^_(2)_=23.048, *p ≤* **0.001**, V=0.327
Psychotic disorders (F20–F29) (n, %)						29	100.0			
	Schizophrenia or -like psychotic disorder					16	55.2			
	Acute psychotic disorder					4	13.8			
	Delusional schizophrenia					2	6.9			
	Psychotic disorder unspecified					4	13.8			
	Major depression with psychotic symptoms					2	6.9			
	Bipolar disorder with psychotic symptoms					1	3.4			
Any CHR criteria										
	APS (n, %)			68	70.1					
	BLIPS (n, %)			2	2.1					
	COPER (n, %)			59	60.8					
	COGDIS (n, %)			37	38.1					

CHR, Clinical High Risk; FEP, First Episode Psychosis; CC, Clinical Controls; SOFAS, Social and Occupational Functioning Assessment Scale of DSM-IV; ISCED, International Standard Classification of Education; OCD, Obsessive-compulsive Disorder; PTBSD, Post Traumatic Stress Disorder; DP/DR, Depersonalization/Derealization; UHR, Ultra-High Risk; BS, Basic Symptoms; APS, Attenuated Positive Symptoms according to SIPS, Structured Interview for Psychosis-Risk Syndrome and/or CAARMS, Comprehensive Assessment of At-Risk Mental States; BLIPS, Brief Limited Intermittent Psychotic Symptoms; COPER, Cognitive-Perceptive Basic Symptoms; COGDIS, Cognitive Disturbances

a Effect sizes reported as Cramer’s V for χ^2^-tests and Fisher’s exact tests; 0.1 equals a small effect, 0.3 a medium effect and 0.5 a large effect.

b FEP vs. CC (z=−3.191, p=0.004, r=0.299, n=114).

c Includes sheltered employment, temporary employment, and regular full- and part-time employment (incl. schooling, academic studies, occupational training, full-time house work).

^*^Standardized cell residuum higher or lower than 1.96. Bold means that the results are significant.

Additionally, the Mini-International Neuropsychiatric Interview for adults (MINI) (47) and its version for children (MINI-Kid) (48) were used to assess diagnoses. Psychosocial functioning was evaluated with the ‘Social and Occupational Functioning Assessment Scale’ (SOFAS) (49).

### MRI Sample 1 of CHR Subjects

We further selected all CHR subjects of sample 1 with available ASL-MRI scans and after artifact rejection (n = 2). In total, of 44 (45.3%) participants (MRI scans were not mandatory) out of 97 CHR subjects, ASL data was available. These 44 CHR subjects were analyzed to investigate differences in rCBF comparing CHR subjects with (n = 21, 21.6%) and without (n = 23, 23.7%) DP/DR symptoms.

### MRI Sample 2 of DP/DR Disorder Patients and Healthy Controls

From six patients (not being part of the other study) with DP/DR disorder according to ICD-10 (five males) aged between 16 and 34 (24.3 ± 7.9 years) and six healthy subjects (three males; 27.0 ± 1.8 years) ASL-MRI scans were analyzed. To assess DP/DR in patients, the CDS (11) was used in the German version, which was found to be reliable (*α* = .95) (50). This self-rating questionnaire consists of 29 items assessing characteristics of depersonalization and derealization (11). Five factors could be extracted from the CDS numbing of emotions, altered body perception, feeling unreal, distorted sense of time, and an unreal seeming environment (51). For each DP/DR experience, the duration and frequency during the last six months were assessed by means of a Likert scale. All patients fulfilled the clinical diagnosis of DP/DR disorder according to ICD-10 (F48.1) assessed through trained interviewers and had CDS scores of 70 ± 39 *(Mdn* = 63), indicating mean to high levels of DP/DR symptoms. A convenience sample of six healthy controls did not met the criteria for any ICD-10 diagnosis. As none of this sample had been suspected to develop psychosis and, therefore, referred to the FETZ Bern, this sample was not examined for CHR criteria.

### MRI Data Acquisition and Processing

The 3.0-Tesla whole-body Siemens MRI system (Magnetom Verio, Siemens Medical Systems, Erlangen, Germany) produced high-resolution structural MRI scans and ASL data in one single session while the subjects were laying alert but with their eyes closed in the MRI. In addition, T1-weighted 3D modified driven equilibrium Fourier transform (MDEFT) images were generated as templates (number of slices, 176; matrix, 256 × 256; slice thickness, 1 mm; voxel size, 1 × 1 × 1 mm^3^) to enable subsequent co-recording of functional data (52). For the pseudo-continuous ASL, interleaved images with and without labeling were obtained in gradient-echo echo-planar imaging sequence (field of view, 220 mm^2^; matrix, 64 × 64; flip angle, 25°; tagging duration, 1,600 ms; post-labeling delay, 1,250 ms; TR/TE, 4,000 ms/13 ms; 100 volumes) (32, 33). The entire brain was contained by the fourteen axonal slices (6 mm thickness and 1.5 mm gap), which were positioned alongside the anterior–posterior commissure line. Matlab (MATLAB and Statistics Toolbox 2012a) and Statistical Parametric Mapping (SPM 8; Wellcome Department of Imaging Neuroscience, London) were used for MRI analysis. The calculation of ASL data was conducted using the aslm toolbox for SPM8 (ASL Imaging Toolbox) (53). Data were visually screened for motion (>3 mm in x, z, or z direction or >3° rotation) and scanner artifacts. Voxelwise mean rCBF for each subject was calculated from flow-time series, subtracting labeled and non-labeled images (54). After realignment and co-registration to the gray matter (GM)-segmented T1 images, normalization was conducted using the SPM Montreal Neurologic Institute T1 template. Spatial smoothing was done with an 8-mm full-width at half maximum kernel. Mean rCBF data were finally normalized [z = (voxel rCBF − global GM rCBF)/SD across individual brain voxels] and GM corrected using GM segments as inclusive masks.

### Statistical Analyses

Behavioral data were analyzed using SPSS (IBM SPSS Statistics for Windows, released 2016, Version 24.0., IBM Corp., Armonk, NY, United States).

For behavior and sample characteristics, frequencies were compared by chi-square tests and continuous or ordinal data with Kruskal–Wallis H tests. Fisher’s exact tests were used when any cells from the chi-square tests contained less than five observations.

For MRI analyses, two-sample t-tests were conducted to compare rCBF between CHR subjects with and without DP/DR (MRI sample 1) and between subjects with DP/DR disorder and healthy controls (MRI sample 2). The results of MRI sample 1 are reported family wise error (FWE), whole brain, corrected at p < 0.05. For MRI sample 2, results are reported following small volume correction for the region of interest (ROI: frontal, temporal and/or striatal areas) and with FWE corrected at p < 0.05. This less conservative approach was used for MRI sample 2 because of its small sample size.

## Results

### Demographics and Psychopathology of Sample 1

The three groups (CHR, FEP, and CC) differed regarding their SOFAS score due to a significantly lower score in FEP compared to CC (see **Table 1**). Further, CHR subjects more often qualified for affective and anxiety disorders and presented more DP/DR symptoms than FEP and CC. There was a significant difference in individuals reporting DP/DR symptoms between CHR and CC (50.5 *vs.* 16.5%; χ^2^_(2)_ = 24.218, *p* ≤ 0.001, Cramer’s V = 0.359) as well as between FEP and CC (37.9 *vs.* 16.5%; χ^2^_(2)_ = 5.960, *p* = 0.015, Cramer’s V = 0.223), indicating moderately higher scores in FEP and CHR as compared to CC (see **Table 1**).

### Demographics of MRI Sample 1

The CHR subjects with DP/DR symptoms differed regarding age, education, and ICD-10 DP/DR disorder from those without DP/DR symptoms (see **Table 2**).

**Table 2:** Sociodemographic and clinical characteristics of clinical high risk (CHR) subjects for psychosis with and without DP/DR with available MRI scans.

		CHR Total		CHR with DP/DR		CHR without DP/DR		Statistical values^a^
		N = 44		n = 21		n = 23		
Age in years								
	mean ± SD	19.8 ± 4.4		20.9 ± 3.9		18.8 ± 4.7		H=4.476, *p*=**0.034**, df=1, ϵ^2 =^ 0.104
	Median	18.4		21.1		17.4		
	Range	13-35		15–26		13–35		
SOFAS score								
	mean ± SD	63.1 ± 10.4		64.7 ± 9.9		61.7 ± 10.9		H=0.830, *p*=0.362, df=1, ϵ^2 =^ 0.019
	Median	65.0		70.0		65.0		
	Range	43–82		48–82		43–75		
Gender, male (n, %)		24	54.5	11	52.4	13	56.5	χ^2^_(1)_ =0.076, *p*=0.783, V=0.042
Current partnership, yes (n, %)		9	20.5	4	19.0	5	21.7	χ^2^_(1)_ =0.212, *p*=0.719, V=0.072
Nationality, Swiss (n, %)		39	88.6	20	95.2	19	82.6	χ^2^_(1)_=1.738, *p*=0.348, V=0.199
Highest education (n, %)								χ^2^_(2)_=13.685, *p ≤* **0.001**, V=0.561
	ISCED 1 (6 school years)	1	2.3	0	0.0	1	4.3	
	ISCED 2 (9–10 school years)	27	61.4	8	38.1	19	82.6	
	ISCED 3 (12–13 school years)	15	34.1	13*	61.9	2*	8.7	
Currently employed or in training/school (n, %)^b^		39	88.6	20	95.2	19	82.6	χ^2^_(1)_=1.738, *p*=0.348, V=0.199
Current alcohol misuse, present (n, %)		3	6.8	2	9.5	1	4.3	χ^2^_(1)_=0.463, *p*=0.599, V=0.103
Current drug misuse, present (n, %)		3	6.8	1	4.8	2	8.7	χ^2^_(1)_=0.267, *p*=1.000, V=0.078
Any current ICD-10 diagnosis (n, %)								
	Any affective disorder (F30–F39)	21	47.7	13	61.9	8	34.8	χ^2^_(1)_=2.968 *p*=0.085, V=0.269
	Any anxiety disorder (F40–F41)	10	22.7	4	19.0	6	26.1	χ^2^_(1)_=0.222, *p*=0.728, V=0.072
	Any eating disorder (F50)	0	0.0	0	0.0	0	0.0	
	OCD (F42)	1	2.3	0	0.0	1	4.3	χ^2^_(1)_=0.934, *p*=1.000, V=0.146
	PTBSD (F43.1)	0	0.0	0	0.0	0	0.0	
	DP/DR disorder (F48.1)	5	11.4	5	23.8	0	0.0	χ^2^_(1)_=6.178, *p*=**0.019**, V=0.375
DP/DR symptoms, present (n, %)		21	47.7	21	100	0	0.0	
DP symptom, present (n, %)		8	18.2	8	38.1	0	0.0	
DR symptoms, present (n, %)		19	43.2	19	90.5	0	0.0	
Any CHR criteria								
	APS syndrome (n, %)	34	77.3	16	76.2	18	78.3	χ^2^_(1)_=0.27, *p*=1.000, V=0.025
	BLIPS syndrome (n, %)	0	0.0	0	0.0	0	0.0	
	COPER (n, %)	29	65.9	15	71.4	14	60.9	χ^2^_(1)_=0.545, *p*=0.460, V=0.111
	COGDIS (n, %)	20	45.5	10	47.6	10	43.5	χ^2^_(1)_=0.076, *p*=0.783, V=0.042

MRI scans were not mandatory for subjects in the CHR group. The MRI subsample was made out of the whole CHR subject group (see **Table 1**) with available MRI scans.

MRI, Magnet Resonance Imaging; CHR, Clinical High Risk; DP/DR, Depersonalization/Derealization; SOFAS, Social and Occupational Functioning Assessment Scale of DSM-IV; ISCED, International Standard Classification of Education; OCD, Obsessive-compulsive Disorder; PTBSD, Post Traumatic Stress Disorder; UHR, Ultra-High Risk; BS, Basic Symptoms; APS, Attenuated Positive Symptoms according to SIPS, Structured Interview for Psychosis-Risk Syndrome and/or CAARMS, Comprehensive Assessment of At-Risk Mental States; BLIPS, Brief Limited Intermittent Psychotic Symptoms; COPER, Cognitive-Perceptive Basic Symptoms; COGDIS, Cognitive Disturbances.

a Effect sizes reported as Cramer’s V for χ^2^-tests and Fisher’s exact tests; 0.1 equals a small effect, 0.3 a medium effect and 0.5 a large effect.

b Includes sheltered employment, temporary employment, and regular full- and part-time employment (incl. schooling, academic studies, occupational training, full-time house work).

^*^Standardized cell residuum higher or lower than 1.96. Bold means that the results are significant.

### Demographics of MRI Sample 2

There were no differences in age (H = 0.232, *p* = 0.630, *ϵ* ^2^ = 0.021) nor sex (χ^2^_(2)_ = 1.500, *p* = 0.545, Cramer’s V = 0.354) between the group with DP/DR disorder and healthy controls.

#### rCBF in MRI Sample 1

**Figure 1:**
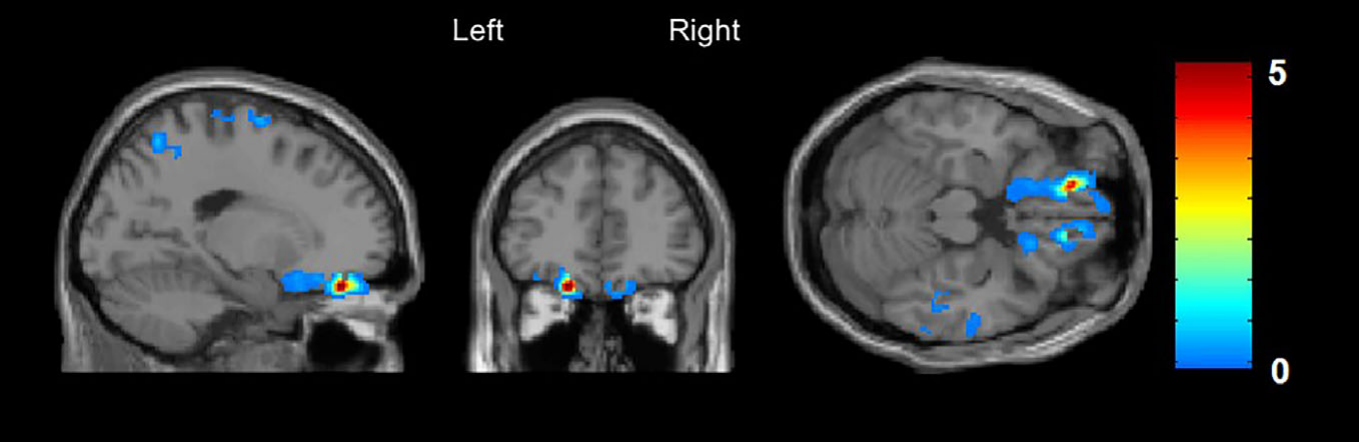
Arterial Spin Labeling analysis for gray matter regional cerebral blood flow (rCBF), whole brain, T-contrast in CHR subjects with (n = 21) *vs.* without (n = 23) DP/DR, uncorrected at p < 0.001 (x/y/z = −16/42/−22, t = 5.3). Red areas indicate significantly decreased CBF in the left orbitofrontal cortex in the CHR group with DP/DR.

A significantly decreased rCBF was found in the left orbitofrontal cortex, Brodmann Area 11, (x/y/z = −16/42/−22, t = 5.3, cluster size = 226, FWE whole brain corrected, p = 0.029) in CHR subjects compared to those without DP/DR symptoms (**Figure 1**).

#### rCBF in MRI Sample 2

**Figure 2:**
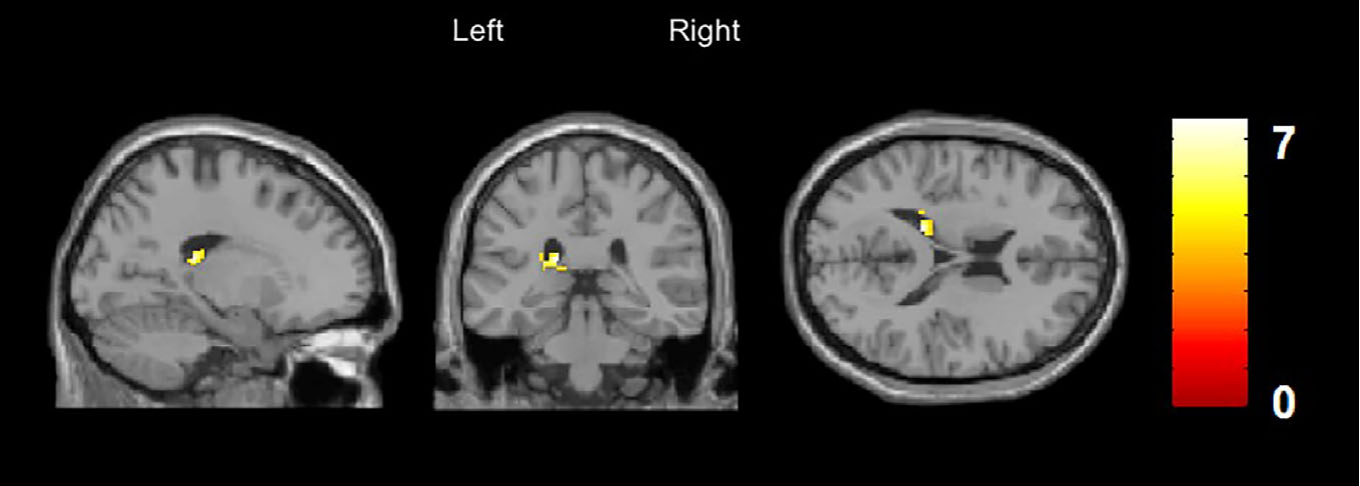
Arterial Spin Labeling Analysis for gray matter regional cerebral blood flow (rCBF), whole brain, T-contrast in DP/DR disorder patients (n = 6) *vs.* healthy controls (n = 6), uncorrected at p < 0.001 (x/y/z = −60/−44/−20, t = 6.7). Yellow areas indicate significantly increased CBF in the left caudate nucleus in DP/DR as compared to controls.

A significantly increased rCBF was discovered in the left caudate nucleus (x/y/z = −18/−32/18, t = 7.6, cluster size = 95) and in the left inferior temporal gyrus (x/y/z = −60/−44/−20, t = 6.7, cluster size = 87) in patients with DP/DR disorder compared to healthy controls. The increase of rCBF in the left caudate nucleus survived FWE (p < 0.05) and small volume correction for the region of interest (**Figure 2**).

## Discussion

This study found a high frequency of DP/DR symptoms in CHR subjects (50.5%) and applied ASL-MRI to investigate DP/DR symptoms for the first time. Using an identical neuroimaging approach, the present study investigates resting state neuronal activity in two different clinical samples, one sample of CHR subjects with a known high prevalence of DP/DR symptoms and an independent sample with DP/DR disorder, exclusively. We found two different brain regions involved with symptoms of DP/DR, namely the orbitofrontal cortex and the caudate nucleus. The left orbitofrontal cortex showed a lower rCBF in CHR subjects with DP/DR symptoms than without, whereas the left caudate nucleus demonstrated a higher rCBF in patients with DP/DR disorder than healthy controls.

The two significant brain regions differ with regard to their structural connections and functions. The orbitofrontal cortex (Brodmann area 11) is heavily connected to the limbic areas, *e.g.* amygdala, hippocampus, and temporal cortex, and the striatum. It receives visual inputs from the temporal cortex and auditory inputs and somatosensory inputs from somatosensory cortical areas and the insula and sends outputs to the temporal cortex, cingulate cortex, and caudate nucleus. The orbitofrontal cortex plays a major role in the computation of expected values and outcome values and their difference and is implicated in positive prediction error signaling *via* dopaminergic neurons in the striatum (55, 56). Other functions of the orbitofrontal cortex are somatosensory integration *e.g.* pleasant/painful touch, visual inputs, *e.g.* face discrimination, reward representation, and cognitive enhancement of the value of affective stimuli (55). Thus, it is important for emotional, perceptional, and cognitive processing-functions that might be disturbed in DP/DR. Moreover, orbitofrontal cortex dysfunction has been implicated in various psychiatric disorders such as borderline personality disorder, posttraumatic stress disorder, major depression, panic disorder or manifest psychosis (57) that can also show DP/DR symptoms (6). Finally, structural and functional findings within the orbitofrontal cortex have been associated with manifest psychosis (58, 59), and psychosis risk findings from the NAPLS study (N = 274 UHR subjects, including 35 converters) indicated that converters experienced a steeper rate of gray matter loss in the medial orbitofrontal cortex (60).

The caudate nucleus is connected with the motoric, sensory, and dorsolateral prefrontal cortex and the lateral orbitofrontal cortex (61, 62). The caudate nucleus is important for the inhibition of motoric impulses and basal learning processes (62) and also for higher cognitive functions including goal-directed actions. It contributes to behavior through the excitation of correct action schemas and the selection of appropriate sub-goals (63) and also to emotions and motivation (64). Dysfunction of the caudate nucleus supported the role as a regulator of fronto-striatal circuits (61) and has been functionally and structurally involved in the pathogenesis of psychosis (65, 66).

Importantly, the orbitofrontal cortex and the caudate nucleus are interconnected and interact with each other through a cortico-striatal circuitry. Top-down and bottom-up processing has become an influential concept in cognitive neuroscience, signifying a highly interactive information exchange where incoming information in lower level sensory regions (*e.g.* auditory input) is modified by higher level cognitive processes (*e.g.* frontal cortex) and *vice versa* (55, 64, 67, 68). The orbitofrontal cortex is involved in top-down and the caudate nucleus in bottom-up processing (69, 70). In predictive coding, sensory perceptions are combined with prior beliefs, thus depending on the successful interaction of bottom-up and top down processes. Prediction errors are defined as the difference between expectations based on the past and the actual outcome (71–74). In psychosis, defective prediction errors may lead to symptoms such as delusions and hallucinations.

Our data suggest that cerebral areas involved in both bottom up and top down processes can be disturbed and therefore lead to DP/DR symptoms. Dysfunctions of the orbitofrontal cortex could change perception “top down” *via* cognition and faulty error prediction in CHR subjects, whereas dysfunctions within the caudate nucleus could change perception “bottom-up” *via* sensory information in patients with DP/DR disorder.

In particular, the caudate nucleus and also the orbitofrontal cortex have previously shown to play a role in DP/DR symptoms (23, 24, 30, 31). Whole brain MRI analyses of gray matter volume in patients with DP/DR and healthy controls showed a decrease in gray matter in the right caudate nucleus that was associated with DP/DR symptom severity (23). Research investigating white matter brain connectivity, using network-based statistics, found a trend supporting the fronto-limbic hypothesis (24). Lower fractional anisotropy in patients with DP/DR than in healthy controls was found in the left caudate nucleus, brainstem, and right amygdala, whereas higher anisotropy was found in the left superior frontal gyrus and right medial orbitofrontal cortex (24). Importantly, one study reported a hypoperfusion of the orbitofrontal cortex (Brodmann area 11) and the left caudate nucleus in a patient group with DP/DR symptoms compared to healthy controls (31). The theory of the fronto-limbic system assumes that the frontal cortex activity is increased (22), but we found a lower rCBF in the orbitofrontal cortex in subjects with DP/DR symptoms. ASL-MRI has many advantages (75–77); however, the nature of cells (excitatory or inhibitory) that contribute to the signal remains unresolved (78). Therefore, brain regions can increase or decrease according to the major cell types (*e.g.* glutamatergic *vs.* GABAergic) that contribute to the signal.

Finally, a PET study reported decrease [11C]raclopride receptor binding potential in the caudate nucleus and putamen bilaterally followed by an increase in the endogenous dopamine availability simultaneously with the emergence of DP/DR symptoms after intake of psilocybin (30).

Hence, imaging studies examining DP/DR reported findings in the caudate nucleus and the orbitofrontal cortex but also mentioned other brain regions, like the temporo-parietal network or the involvement of the limbic system (21, 28). Our own data did not clearly demonstrate changes in the temporo-parietal network or the limbic system. The lack of the involvement of the temporo-parietal network might be explained by the different symptoms of DP/DR. Feelings of disembodiment and lack of agency are explained by this system (21, 79), whereas emotional numbing and perceptual detachment are explained by the fronto-limbic system. This study did not differentiate the type of DP/DR to test those systems.

Several disorders show symptoms of DP/DR like posttraumatic stress disorder, depression, anxiety (1, 7) but also in psychosis (12, 14) and in our study, in CHR subjects. In patients clinically suspected to develop psychosis, DR as well as both auto- and somatopsychic DP occurred in roughly 16% of cases, but only DR was found to be psychosis-predictive (46, 80). An explanation of the DP/DR symptoms in psychosis is that an impairment of multisensory integration could lead to incoherent self-experiences, which then leads to DP/DR. It is hypothesized that the brain’s efforts to fix that perceptual incoherence could result in hallucinations and delusions because the focus lies on the DP/DR and no longer on the real outside world (81); therefore, DP/DR was also considered as a predelusional state (82). Self-disturbances in CHR subjects, such as DP/DR, might be potential markers of psychosis (83–85). Self-disturbances are generally described as anomalies of subjective experiences such as disruption of the stream of consciousness, distortion of sense of presence, corporeality or difficulties in self-demarcation (83). Self-disturbances or self-disorders are conceptualized as a constellation of interrelated anomalies of subjective experience gravitating around pervasive distortions of the “minimal” or “core self” (86). Self-disturbances are usually self-recognized in CHR subjects due to intact self-monitoring, whereas in patients transitioning to psychosis those disturbances are no longer recognized.

Self-disturbances can be measured with the Examination of Anomalous Self-Experience scale (87) or through some of the basic symptoms as they are defined as self-experienced disturbances (15, 86).

DP/DR symptoms were found to be more frequent, had a longer duration, and were stronger in the early stages of psychosis than in the chronic stages. Therefore, it was proposed that the symptoms would predate the onset of psychosis as they were more often in the early stages than in the chronic ones (8). The orbitofrontal cortex and caudate nucleus have also been associated with psychosis (58, 59, 65, 66). Therefore, in future longitudinal studies, the role of DP/DR symptoms and involved brain regions (orbitofrontal cortex and caudate nucleus) with regard to a conversion to psychosis should be investigated. With this, the potential value of DP/DR symptoms as an additional predictor for psychosis in CHR subjects could be further evaluated.

### Strengths and Limitations

One strength of this study is the investigation of two different samples with the same method. The first sample with CHR subjects with or without DP/DR symptoms and the second sample with DP/DR disorder and healthy controls. The ASL-MRI signal is directly linked to resting-state rCBF and provides a quantitative and absolute measure of rCBF, reflecting the level of neuronal activity (34, 35) plus providing the potential to measure changes in striatal neuronal activity (88–90).

Despite the strengths of our study, some limitations must be considered. The small sample size with DP/DR disorder is a major limitation. Further studies should involve larger samples to increase the statistical power of the analyses. However, we still wanted to highlight the findings from this small sample as we believe the inclusion of this group is of scientific value. The majority of studies with DP/DR using MRI investigated DP/DR symptoms in disorders such as major depression, posttraumatic stress disorder, drug abuse, or borderline personality disorder, whereas this study investigated pure DP/DR disorder without comorbidities. Pure DP/DR disorder patients without concomitant additional diagnoses are difficult to find and hard to motivate for study participation. With regard to sample size, several published studies are comparable to our study [*e.g.* (27, 91)]. Another limitation is the classification of DP/DR. In sample 1, DP/DR was narrowly assessed in a clinical interview through the SPI-CY/SPI-A and transformed into a binary variable. In the SPI-A the autopsychic DP was not collected, whereas in the SPI-CY it was. That could lead to a small bias of CHR with and without DP/DR symptoms. In sample 2, DP/DR was more broadly assessed, and participants were grouped into DP/DR or healthy controls. Because DP/DR is reported to lie on a continuum, analyses would ideally involve DP/DR on an ordinal scale.

There was no differentiation in symptoms of DP/DR in this study. Despite that they are subsumed as DP/DR, future studies should differentiate the symptoms of DP/DR in different subgroups as this could give more comprehension in the neurobiological correlations and consider the comorbidities and developmental aspects such as age. A further limitation is the direct comparison of the two samples in this study as they were not assessed with the exact same clinical instruments. Future studies should use the same instruments to detect psychiatric disorders and CHR symptoms as well as DP/DR. With the CDS DP/DR could be assessed on a scale and differentiated, and other disorders or CHR symptoms could be used as covariates.

### Conclusion

To summarize, DP/DR symptoms are frequent in CHR subjects. Investigating two separate DP/DR populations with an identical neuroimaging technique, we found decreased neuronal activity in the orbitofrontal cortex but increased activity in the caudate nucleus.

As the orbitofrontal cortex is involved with psychiatric disorders that are associated with DP/DR symptoms (1, 7, 57), we conclude that the area is important for the emergence of DP/DR. According to its function in somatosensory integration, it is reasonable that DP/DR can be seen as a failure in somatosensory integration, which could turn into numbing of emotions, altered body perception, feeling unreal, distorted sense of time, or perceiving an unreal environment.

The caudate nucleus as part of the striatum is connected to prefrontal areas (36) and is important for cognitive functions, emotions, and motivation and could play a role in the control function from fronto-limbic system and therefore produce DP/DR symptoms.

Our results indicate that there seem to be divergent mechanisms that finally lead to the same/similar symptoms. This suggests that top-down (orbitofrontal cortex) and bottom-up (caudate nucleus) mechanisms might contribute to a different extent to the emergence of DP/DR, depending on the manifestation/phenomenology of the symptoms.

## Data Availability Statement

The datasets generated for this study are available on request to the corresponding author.

## Ethics Statement

All procedures contributing to this work comply with the ethical standards of the relevant national and institutional committees on human experimentation and with the Helsinki Declaration of 1975, as revised in 2013. The human research ethics committee of the Canton Bern approved the study (ID PB_2016-01991, KEK-095/10). All participants gave written informed consent, and in minors, parental written informed consent was provided to participate in this study.

## Author Contributions

JB wrote the first draft of the manuscript and did the statistical analysis. CM and JK rewrote sections of the manuscript. AF and MH provided MRI knowledge. SK and BS were responsible for the design of the DP/DR disorder sample. FS-L was leader of the FETZ. FS-L, DH, SW, and MK gave meaningful inputs for the manuscript. All authors contributed to the article and approved the submitted version.

## Funding

This study was supported by internal funding of the University Hospital of Child and Adolescent Psychiatry and Psychotherapy, the University Hospital of Psychiatry and Psychotherapy being part of the University of Bern, and the Soteria Bern.

## Conflict of Interest

BS has been a consultant and/or advisor to, or has received honoraria from AstraZeneca, Bristol-Myers Squibb, Eli Lilly, Janssen, Novartis, and Shire.

The remaining authors declare that the research was conducted in the absence of any commercial or financial relationships that could be construed as a potential conflict of interest.
