# Supplementary material for: Trapped in a Glass Bell Jar: Neural Correlates of Depersonalization and Derealization in Subjects at Clinical High-Risk of Psychosis and Depersonalization–Derealization Disorder
Source: Front Psychiatry. 2020 Sep 11;11:535652. doi: 10.3389/fpsyt.2020.535652 (PMC7516266; doi:10.3389/fpsyt.2020.535652)
Supplement: Supplementary file 1 [file DataSheet_1.docx]

**Supplementary Material**

Trapped in a glass bell jar: Neural correlates of depersonalization and derealization in subjects at clinical high-risk of psychosis and depersonalization-derealization disorder

Büetiger et al.

| **I. Supplementary Text S1:** | *Definition of derealization and depersonalization.* |
| --- | --- |
|  |  |
|  |  |
|  |  |
|  |  |
|  |  |
|  |  |
|  |  |
|  |  |
|  |  |
|  |  |

**I. Supplementary Text S1.** *Definition of derealization and depersonalization.*

Derealization and depersonalization are described in the Schizophrenia Proneness Instruments Children & Youth and Adult (SPI-CY and SPI-A) as follows (Schultze-Lutter, Addington, Ruhrmann, & Klosterkötter, 2007; Schultze-Lutter, Marshall, & Koch, 2012):

**Derealization**

- **SPI-CY: derealization (item B7)**
- **SPI-A: derealization (item O8)**

Derealization is defined as a change in how one relates emotionally to the environment. This can be either in the form of an estrangement and detachment from the visual world, i.e., from how one sees the world or an increased emotional affinity for the environment. In the first, the environment appears unreal, altered and strange in a way that is often hard to describe and is not due to actual perceptual changes or illusions or delusional misidentifications. The person feels estranged from his or her own world. The usual emotional ties to his or her surroundings no longer exist or have become weaker and the person feels disconnected from the world around him/her. The latter describes that the world or certain aspects of it are exceptionally emotional impressive. This experience often occurs in tandem with a captivation by details of perception. This kind of derealization is often accompanied by positive feelings or even euphoria.

**Depersonalization**

- **SPI-CY: somatopsychic bodily depersonalization (item B8.2) and autopsychic depersonalization (item C6)**
- **SPI-A: somatopsychic bodily depersonalization (item F6)**

Somatopsychic depersonalization consists of a feeling of alienation from one’s own body or parts of it. The body or parts of it are perceived as not belonging to oneself anymore. A part of the body may seem to be isolated or separated from the rest, or may feel as if it does not exist at all. Another variety of this symptom is a sensation of fragmentation in which the body feels like it is falling apart or body parts feel like they are no longer connected, even though all parts are still experienced as belonging to the person, (i.e., are not alienated). Autopsychic depersonalization consists of feeling alienated or detached from one’s own thoughts, emotions and behavior. Whilst talking, the person listens to himself or herself as if listening to someone else speaking. The person’s thoughts and emotions appear to be peculiarly weak, insipid or lacking in substance or his or her thoughts and actions appear somehow unreal, remote or automatic. To be rated, the person must have insight into the experience (e.g., that their thoughts are still their own) and the experience should not be due to perceptual disturbances a decreased ability to experience or express emotions. Furthermore, the experience should not be confined to situations of great fatigue, severe and persistent overstrain or deep emotional distress.

**Time span**

Both derealization and depersonalization are rated in the SPI-CY and SPI-A within the last three months.

**References**

Schultze-Lutter, F., Addington, J., Ruhrmann, S., & Klosterkötter, J. (2007). *Schizophrenia proneness instrument, adult version (SPI-A)*. Rome: Fioriti.

Schultze-Lutter, F., Marshall, M., & Koch, E. (2012). *Schizophrenia Proneness Instrument, Child and Youth (SPI-CY) - Extended English Translation (EET)*. Retrieved from https://www.fioritieditore.com/prodotto/schizophrenia-proneness-instrument-child-and-youth-spi-cy-extended-english-version-2/
